# Histological Analysis of Arterial and Venous Grafts Used in Coronary Bypass for Patients With Renal Insufficiency: A Prospective Multicentre Observational Study

**DOI:** 10.1093/icvts/ivaf222

**Published:** 2025-09-24

**Authors:** Valentina Grazioli, Michele Di Mauro, PierSilvio Gerometta, Barbara Parrella, Matteo Matteucci, Andrea Musazzi, Mauro Rinaldi, Marta Sannito, Paolo Panisi, Alfonso Agnino, Elizabeth Boulos Issa Sweidan, Debora Guareschi, Mario Gaudino, Domenico Corradi, Roberto Lorusso

**Affiliations:** Cardio-Thoracic Surgery Department, Maastricht University Medical Centre, Maastricht, 6229 ER Maastricht, The Netherlands; Cardiovascular Research Institute Maastricht (CARIM), Maastricht, 6229 ER Maastricht, The Netherlands; Department of Cardiac Surgery, Humanitas Gavazzeni Clinic, 24125 Bergamo, Italy; Cardio-Thoracic Surgery Department, Maastricht University Medical Centre, Maastricht, 6229 ER Maastricht, The Netherlands; Cardiovascular Research Institute Maastricht (CARIM), Maastricht, 6229 ER Maastricht, The Netherlands; Department of Cardiac Surgery, Humanitas Gavazzeni Clinic, 24125 Bergamo, Italy; Division of Cardiac Surgery, Cardiovascular and Thoracic Department, Molinette Hospital—Città della Salute e della Scienza, University of Turin, 10126 Turin, Italy; Cardiac Surgery Unit, Ospedale di Circolo, 21100 Varese, Italy; Cardiac Surgery Unit, Ospedale di Circolo, 21100 Varese, Italy; Division of Cardiac Surgery, Cardiovascular and Thoracic Department, Molinette Hospital—Città della Salute e della Scienza, University of Turin, 10126 Turin, Italy; Department of Cardiac Surgery, Humanitas Gavazzeni Clinic, 24125 Bergamo, Italy; Division of Cardiac Surgery, Foundation I.R.C.C.S. Policlinico San Matteo, 27100 Pavia, Italy; Department of Cardiac Surgery, Humanitas Gavazzeni Clinic, 24125 Bergamo, Italy; Division of Robotic and Minimally Invasive Cardiac Surgery, Department of Cardiovascular Surgery, Cliniche Humanitas Gavazzeni, 24125 Bergamo, Italy; Department of Medicine and Surgery, Unit of Pathology, University of Parma, 43100 Parma, Italy; Department of Medicine and Surgery, Unit of Pathology, University of Parma, 43100 Parma, Italy; Department of Cardiothoracic Surgery, Weill Cornell Medicine, Presbyterian Hospital, NY 10065, United States; Department of Medicine and Surgery, Unit of Pathology, University of Parma, 43100 Parma, Italy; Cardio-Thoracic Surgery Department, Maastricht University Medical Centre, Maastricht, 6229 ER Maastricht, The Netherlands; Cardiovascular Research Institute Maastricht (CARIM), Maastricht, 6229 ER Maastricht, The Netherlands

**Keywords:** chronic kidney disease, coronary artery bypass grafting, graft histology, intimal thickening, fibroelastosis

## Abstract

**Objectives:**

Chronic kidney disease (CKD) is associated with metabolic dysfunctions that accelerate atherosclerosis, posing significant challenges for patients undergoing coronary artery bypass grafting (CABG). In this population, arterial calcification and reduced saphenous vein patency are common complications. This multicentre prospective study aims to evaluate the impact of renal dysfunction on the histological characteristics of arterial and venous grafts used in CABG.

**Methods:**

Vascular graft specimens collected during CABG were prospectively analysed and stratified into 3 groups based on renal function, according to established publications: Group 1 (glomerular filtration rate [GFR] ≥90 mL/min/1.73 m^2^), Group 2 (GFR 60-89 mL/min/1.73 m^2^), and Group 3 (GFR ≤59 mL/min/1.73 m^2^). Formalin-fixed samples were histologically assessed for intimal thickening (Grade 0-3), fibroelastosis, and vasa vasorum density.

**Results:**

A total of 324 arterial grafts (Group 1: 100; Group 2: 134; Group 3: 90) and 289 vein grafts (Group 1: 86; Group 2: 119; Group 3: 84) were analysed, including 5 arterial and venous grafts from dialysis patients. No significant structural differences were observed between groups. Intimal thickening rates were comparable across renal function stages. Fibroelastosis was more prevalent in venous grafts (56%-64%) than in arterial grafts (10%-15%).

**Conclusions:**

This study assesses graft histology in CABG patients stratified by renal function. At surgery, CKD does not appear to significantly alter graft structure. Further studies are warranted to explore long-term graft outcomes in this population.

## Graphical abstract

**Figure:**
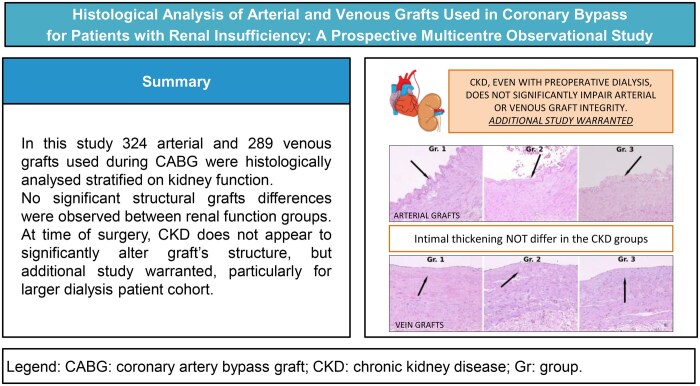

## INTRODUCTION

Chronic kidney disease (CKD) is a well-recognized risk factor for cardiovascular disease and is present in approximately a quarter of patients undergoing coronary artery bypass grafting (CABG).^1^^,^^2^ CKD contributes to vascular dysfunction and exhibits distinct features across arterial and venous compartments.^3–6^ The internal thoracic artery (ITA) and saphenous vein grafts (SVGs) are the most used conduits in CABG, regardless of kidney function. In arteries, CKD is associated with an accelerated, aggressive atherosclerosis, with marked calcification in both intimal and medial layers.^5^^,^^7^ Notably, ITA, known for its resistance to atherosclerosis, appears to maintain this resilience even in CKD.^8–10^

SVGs show reduced patency with worsening CKD, due to a more aggressive degeneration of the venous system.^1^^,^^8^^,^^10–15^ Moreover, SVGs are particularly susceptible to thrombosis, driven by arterialization post-implantation and the pro-inflammatory CKD milieu, which impairs endothelial function and graft integrity.^13–16^

In the current literature, evidence on whether ITA and SVG grafts may already exhibit damage prior to surgery due to CKD is limited. Most evidence on graft patency comes from postoperative angiography or postmortem histology.^3^^,^^8^^,^^17–20^ Few studies have explored graft histology intraoperatively or during reintervention,^9^^,^^15^^,^^21–24^ and only Ura and Kinoshita included CKD or dialysis patients. Our study aims to address this gap by investigating pre-surgical vascular changes associated with CKD. Therefore, we conducted a multicentre prospective study to evaluate baseline graft histology at CABG surgery and to determine whether CKD induces structural alterations proportional to disease severity.

## MATERIALS AND METHODS

### Ethical statement

The Ethics Committee of Humanitas Research Hospital, Rozzano, approved this study (Protocol GAV 546/20) on July 20, 2020. All participants were aged 18 years or older. Written informed consent was obtained from each participant prior to enrolment in the study. All data were anonymized. Data and biological materials were collected and stored solely for the purposes of the present study, with approval from the Ethics Committee of Humanitas Research Hospital, Rozzano.

### Study population

We analysed data from consecutive patients undergoing isolated CABG (on- or off-pump) between July 2020 and March 2024 at 3 Italian Centers: Humanitas Gavazzeni Hospital (Bergamo), ASST dei Sette Laghi Hospital (Varese), and Molinette Hospital—Città della Salute e della Scienza (Turin). Informed consent for surgery was obtained. Discarded graft specimens were prospectively collected. Preoperative, intraoperative, postoperative, and histological data were recorded in a dedicated electronic database.

Renal function was assessed using the glomerular filtration rate (GFR) calculated with the Cockcroft-Gault equation. According to the 2017 KDIGO guidelines,^25^ patients were staged from G1 (GFR ≥ 90 mL/min/1.73 m^2^) to G5 (GFR < 15 mL/min/1.73 m^2^). For analytical purposes, we adopted the classification by Milojevic et al.^26^ Group 1: patients with normal renal function (Stage G1); Group 2: patients with impaired renal function (Stage G2); Group 3: CKD (Stages G3, G4, and G5).

A subgroup analysis was specified exclusively for Group 3 to compare patients with a GFR ≤59 mL/min versus those on dialysis and patients with a GFR of 58-31 mL/min versus those with a GFR ≤30 mL/min.

ITAs were harvested either skeletonized or pedicled, and SVGs via the open technique. Discarded ITA (distal) and SVG (cranial) segments were collected immediately after graft preparation, placed in sterile pre-labelled containers with 10% neutral buffered formalin, and fixed at room temperature for ≥24 h (≥10× tissue volume). Samples were then paraffin-embedded for histology using standardized protocols across all centres.

### Histological analysis

All vascular specimens were analysed at the Department of Pathological Anatomy, University of Parma. Paraffin-embedded samples were sectioned at 3 µm and stained with haematoxylin and eosin. Intimal thickening was defined as subendothelial fibrous proliferation. A semi-quantitative scale—which was chosen to avoid biases in terms of non-perpendicular cutting planes of the histological sections—assessed its thickness relative to that of the tunica media: Grade 0 (none); Grade 1 (<20%); Grade 2 (20%-40%); Grade 3 (>40%). Fibroelastosis was defined as an accumulation, even if focal, of fibrous and elastic fibres within the vascular wall and reported as presence/absence. Vasa vasorum density was quantified in representative fields at 20× magnification (area: 2 mm^2^; diameter: 1.6 mm) and expressed as mean/20× histological field.

Venous arterialization—a series of morpho-functional changes occurring when a vein segment is transferred as a bypass graft into the arterial circulation—consisted of intimal hyperplasia, tunica media thickening, and wall deposition of elastic fibres along with creation of an internal elastic lamina.^27^ The explored histopathological modifications are pictorially shown in **Figures S1 and S2**.

### Statistical analysis

Categorical data were reported as count and percentage; continuous data were reported as median and quartiles. Comparison among the 3 groups was performed using the Kruskal-Wallis rank sum test in case of continuous data; Pearson’s chi-squared test was used in case of categorical data. *P*-value below .05 was considered statistically significant. R Core Team (2024). R: A Language and Environment for Statistical Computing. R Foundation for Statistical Computing, Vienna, Austria (https://www.R-project.org/).

## RESULTS

### Histological findings

**Figure 1.**
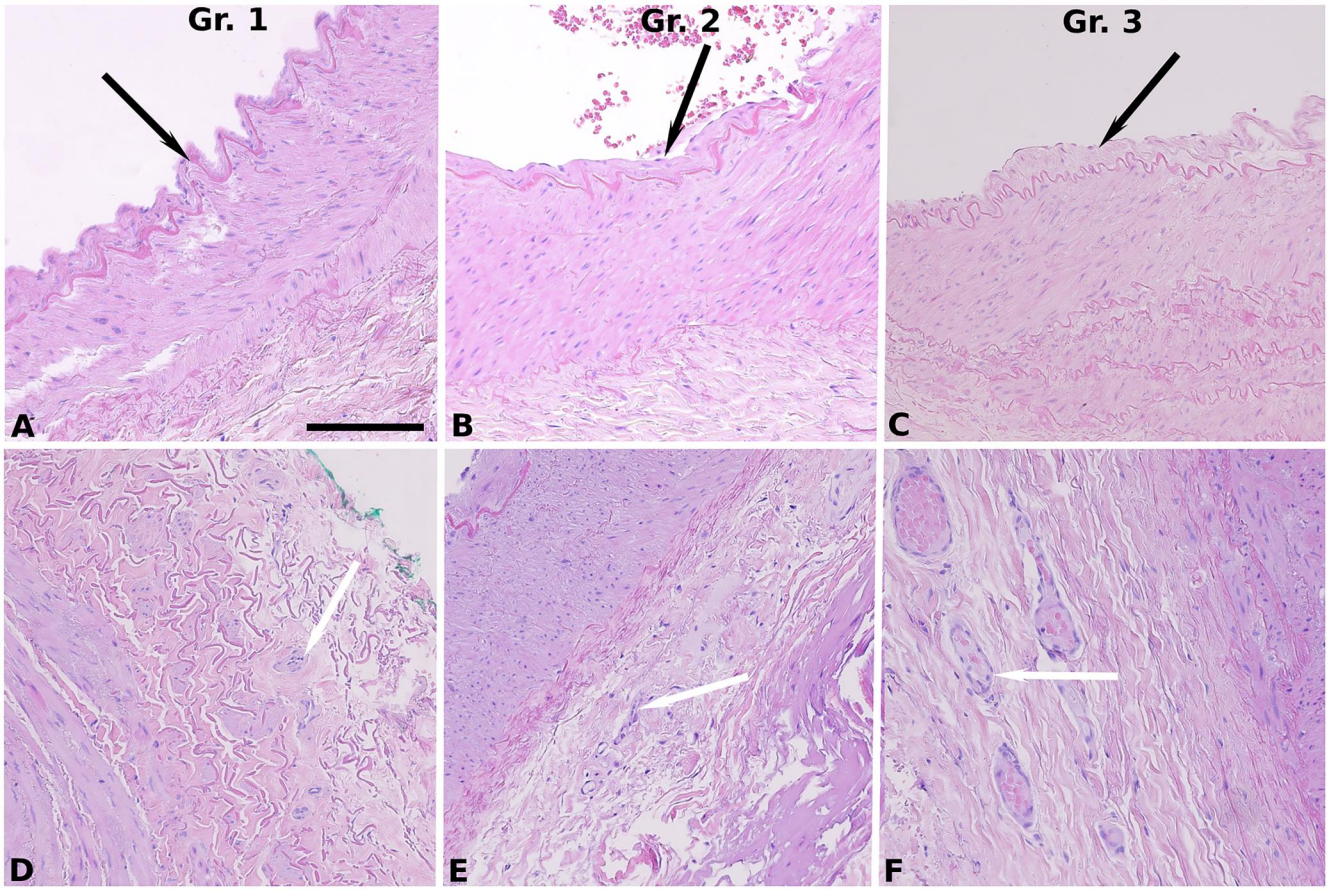
Histopathology of Arterial Grafts. (A-C) Similar intimal thickening (Grades 1 and 2) across Groups 1-3. (D-F) Comparable vasa vasorum density across Groups 1-3. Black arrows = intimal thickening; white arrows = adventitial vasa vasorum; Gr. = group. Staining: haematoxylin-eosin; magnification: 20× (scale bar is 150 µm)

**Figure 2.**
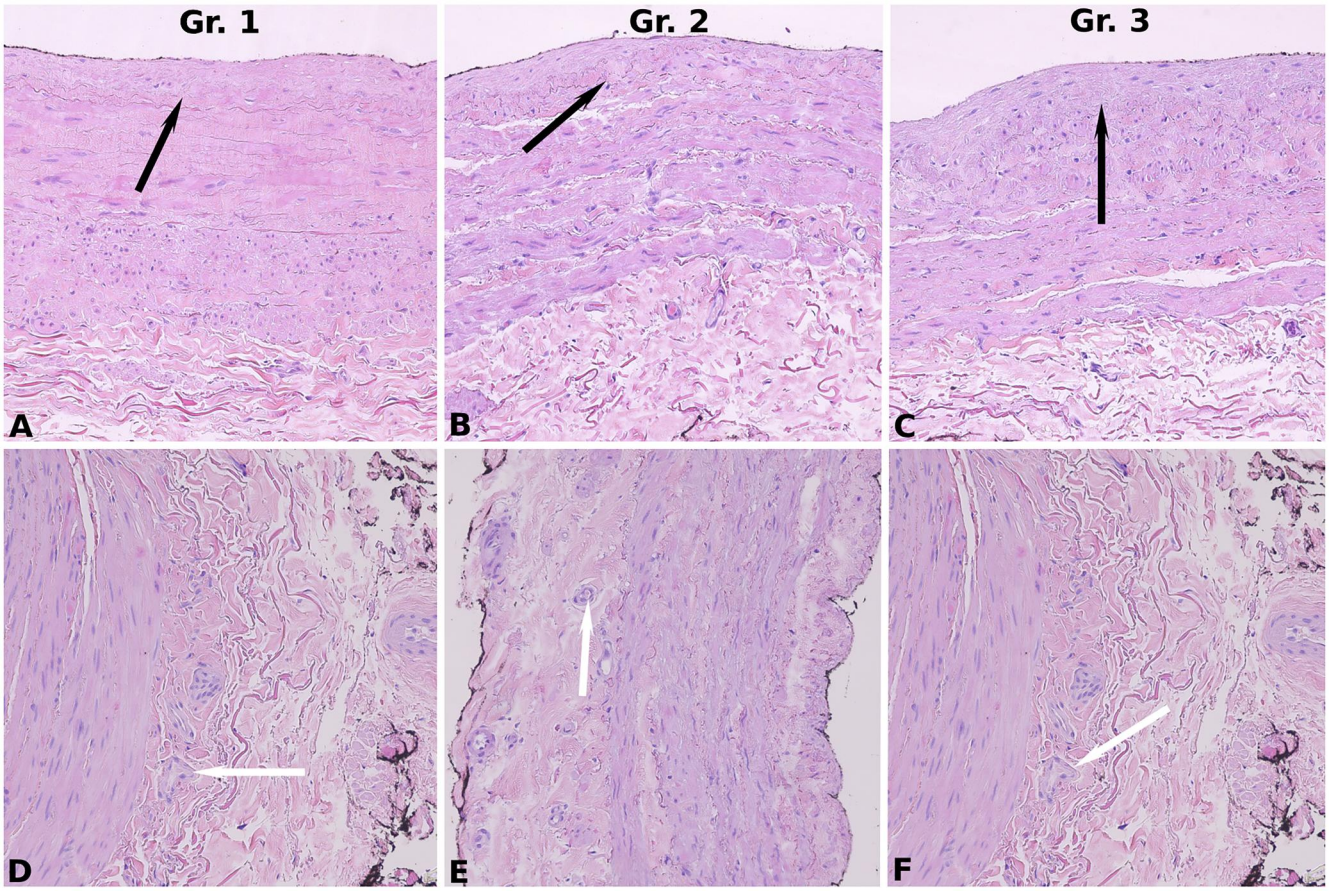
Histopathology of Venous Grafts. (A-C) Similar intimal thickening (Grade 1) across Groups 1-3. (D-F) Comparable vasa vasorum density across Groups 1-3. Black arrows = intimal thickening; white arrows = adventitial vasa vasorum; Gr. = group. Staining: haematoxylin-eosin; magnification: 20× (scale bar is 150 µm)

Discarded ITA segments were collected from 323 patients (338 arterial grafts, including bilateral ITA cases). Of these 338 grafts, 14 were excluded due to insufficient tissue volume. SVG segments were obtained from 289 patients (289 analysable specimens). Histological characteristics are depicted in **Figures 1 and 2** and **Table 1**.

**Table 1.** Histological Characteristics of the ITA and SVG Grafts

ITA grafts				
Characteristic	Group 1	Group 2	Group 3	*P*-value^b^
	(*N* = 100)^a^	(*N* = 141)^a^	(*N* = 95)^a^	
Arterial specimens				0.2
0, *n* (%)	1 (1.0%)	7 (5.0%)	5 (5.3%)	
1, *n* (%)	98 (98%)	134 (95%)	90 (95%)	
2, *n* (%)	1 (1.0%)	0 (0%)	0 (0%)	
Vasa vasorum, *n* (IQR)	7 (5, 10)	7 (5, 10)	8 (5, 11)	0.8
Intimal thickening				0.2
None, *n* (%)	65 (68%)	79 (62%)	62 (73%)	
Grade 1, *n* (%)	21 (22%)	38 (30%)	20 (24%)	
Grade 2, *n* (%)	10 (10%)	9 (7.1%)	2 (2.4%)	
Grade 3, *n* (%)	0 (0%)	1 (0.8%)	1 (1.2%)	
Fibroelastosis, *n* (%)	10 (10%)	16 (13%)	13 (15%)	0.6

SVG grafts				
Characteristic	Group 1	Group 2	Group 3	*P*-value^b^
	(*N* = 100)^a^	(*N* = 141)^a^	(*N* = 95)^a^	
Venous specimens, *n* (%)	86 (86%)	119 (85%)	84 (88%)	0.8
Vasa vasorum, *n* (IQR)	9 (6, 12)	9 (6, 11)	10 (7, 12)	0.5
Intimal thickening				0.2
None, *n* (%)	13 (16%)	14 (12%)	15 (19%)	
Grade 1, *n* (%)	36 (46%)	47 (42%)	44 (56%)	
Grade 2, *n* (%)	17 (22%)	27 (24%)	10 (13%)	
Grade 3, *n* (%)	13 (16%)	24 (21%)	10 (13%)	
Fibroelastosis, *n* (%)	47 (59%)	63 (56%)	51 (64%)	0.6
Vein arterialization, *n* (%)	16 (20%)	27 (24%)	25 (31%)	0.3

a *n* (%); median (IQR).

b Fisher’s exact test; Pearson’s chi-squared test; Kruskal-Wallis rank sum test.

Abbreviations: ITA, internal thoracic; *N*, number of patients; SVG, saphenous vein grafts.

Intimal thickening did not significantly differ across kidney function groups for ITA (*P* = .2) or SVG (*P* = .2). Most ITAs showed no intimal thickening (68% Group 1, 62% Group 2, and 73% Group 3), while Grade 1 thickening predominated in SVGs (46% Group 1, 42% Group 2, and 56% Group 3.) Fibroelastosis was rare in ITAs (10%-15%) but prevalent in SVGs (59%-64%), with no significant renal group differences. Vein arterialization also showed no significant variation (*P* = .3). Vasa vasorum counts in the ITA were similar across renal groups; SVGs showed higher vasa vasorum prevalence than ITAs (**Table 1**).

Subgroup analysis within Group 3 (**Tables 2 and 3**) revealed no significant differences in ITA histology between patients with GFR ≤59 mL/min and those on dialysis, or between those with GFR 58-31 mL/min and ≤30 mL/min. ITAs mostly showed no intimal thickening and minimal fibroelastosis. SVGs showed uniform findings—Grade 1 thickening, fibroelastosis >50%, and comparable vasa vasorum and arterialization—without subgroup differences.

**Table 2.** Histological Characteristics of ITA Grafts in Group 3, Analysed by Subgroups

Characteristic	GFR ≤ 59 mL/min	Dialysis	***P*-value** ^b^
	**(*N* = 89)** ^a^	**(*N* = 6)** ^a^	
Arterial specimens, *n* (%)	85 (96%)	5 (83%)	0.3
Vasa vasorum, *n* (IQR)	8 (5, 11)	5 (5, 8)	0.7
Intimal thickening			0.6
None, *n* (%)	58 (72%)	4 (100%)	
Grade 1, *n* (%)	20 (25%)	0 (0%)	
Grade 2, *n* (%)	2 (2.5%)	0 (0%)	
Grade 3, *n* (%)	1 (1.2%)	0 (0%)	
Fibroelastosis, *n* (%)	13 (16%)	0 (0%)	>0.9

Characteristic	GFR 58-31 mL/min	GFR ≤ 30 mL/min	*P*-value^b^
	(*N* = 79)^a^	(*N* = 16)^a^	
Arterial specimens, *n* (%)	76 (96%)	14 (88%)	0.2
Vasa vasorum, *n* (IQR)	8 (6, 11)	5 (5, 9)	0.1
Intimal thickening			0.7
None, *n* (%)	51 (71%)	11 (85%)	
Grade 1, *n* (%)	18 (25%)	2 (0%)	
Grade 2, *n* (%)	2 (2.8%)	0 (0%)	
Grade 3, *n* (%)	1 (1.4%)	0 (0%)	
Fibroelastosis, *n* (%)	13 (18%)	0 (0%)	0.2

a *n* (%); median (IQR).

b Fisher’s exact test; Pearson’s chi-squared test; Kruskal-Wallis rank sum test.

Abbreviations: GFR, glomerular filtration rate; ITA, internal thoracic artery; *N*, number of patients.

**Table 3.** Histological Characteristics of Venous Grafts in Group 3, Analysed by Subgroups

Characteristic	GFR ≤ 59 mL/min	Dialysis	***P*-value** ^b^
	**(*N* = 89)** ^a^	**(*N* = 6)** ^a^	
Venous specimens, *n* (%)	79 (89%)	5 (83%)	0.5
Vasa vasorum, *n* (IQR)	9 (7, 12)	12 (11, 13)	0.12
Intimal thickening			0.7
None, *n* (%)	14 (19%)	1 (20%)	
Grade 1, *n* (%)	42 (57%)	2 (40%)	
Grade 2, *n* (%)	9 (12%)	1 (20%)	
Grade 3, *n* (%)	9 (12%)	1 (20%)	
Fibroelastosis, *n* (%)	49 (65%)	2 (40%)	0.3
Vein arterialization, *n* (%)	23 (31%)	2 (40%)	0.6

Characteristic	GFR 58-31 mL/min	GFR ≤ 30 mL/min	*P*-value^b^
	(*N* = 79)^a^	(*N* = 16)^a^	
Venous specimens, *n* (%)	70 (89%)	14 (88%)	>0.9
Vasa vasorum, *n* (IQR)	9 (7, 11)	12 (7, 13)	0.07
Intimal thickening			0.4
None, *n* (%)	11 (17%)	4 (31%)	
Grade 1, *n* (%)	39 (59%)	5 (38%)	
Grade 2, *n* (%)	8 (12%)	2 (15%)	
Grade 3, *n* (%)	8 (12%)	2 (15%)	
Fibroelastosis, *n* (%)	44 (66%)	7 (54%)	0.5
Vein arterialization, *n* (%)	21 (31%)	4 (31%)	>0.9

a *n* (%); median (IQR).

b Fisher’s exact test; Pearson’s chi-squared test; Kruskal-Wallis rank sum test.

Abbreviations: GFR, glomerular filtration rate; *N*, number of patients.

### Patients characteristics and clinical outcomes

We included 336 patients: 100 in Group 1, 141 in Group 2, and 95 in Group 3 (**Table 4**). Median age was highest in Group 3 (75 [69-79] versus 69 [64-73] and 63 [57-67] in Groups 2 and 1, *P* < .001). Creatinine was elevated and GFR reduced in Group 3 compared with Groups 1 and 2 (*P* < .001). Group 3 also had lower weight and higher EuroSCORE II (*P* < .001). Other baseline characteristics did not differ significantly (**Table 4**).

**Table 4.** Preoperative Baseline Characteristics

Characteristic	Group 1	Group 2	Group 3	*P*-value^b^
	(*N* = 100)^a^	(*N* = 141)^a^	(*N* = 95)^a^	
Age, years	63 (57, 67)	69 (64, 73)	75 (69, 79)	<0.001
Sex				0.2
M, *n* (%)	83 (83%)	123 (87%)	75 (79%)	
Weight, kg	84 (74, 90)	75 (68, 81)	74 (65, 80)	<0.001
Height, m	1.72 (1.67, 1.76)	1.70 (1.65, 1.75)	1.70 (1.63, 1.75)	0.094
Creatinine, mg/dL	0.85 (0.75, 0.95)	0.98 (0.90, 1.10)	1.44 (1.17, 1.77)	<0.001
GFR, mL/min	100 (95, 113)	75 (66, 81)	48 (37, 55)	<0.001
Smoke				0.006
No smoker, *n* (%)	50 (51%)	87 (64%)	55 (59%)	
Active smoker, *n* (%)	26 (26%)	14 (10%)	10 (11%)	
Previous smoker, *n* (%)	23 (23%)	36 (26%)	29 (31%)	
Diabetes, *n* (%)	33 (33%)	50 (36%)	37 (39%)	0.7
PAD, *n* (%)	9 (9.1%)	11 (8.0%)	15 (16%)	0.13
Carotid sclerosis, *n* (%)	12 (12%)	33 (24%)	20 (22%)	0.066
COPD, *n* (%)	5 (5.1%)	5 (3.6%)	8 (8.5%)	0.3
HTA, *n* (%)	73 (74%)	99 (72%)	76 (81%)	0.3
Dyslipidaemia, *n* (%)	53 (54%)	84 (62%)	60 (64%)	0.3
EF, %	55 (50, 60)	55 (50, 59)	55 (44, 60)	0.13
ACS, *n* (%)	38 (38%)	52 (38%)	35 (37%)	>0.9
Previous MI, *n* (%)	13 (13%)	21 (15%)	22 (23%)	0.13
EuroSCORE, %	0.90 (0.69, 1.11)	1.11 (0.85, 1.54)	2.38 (1.69, 3.60)	<0.001
Number of CABG				
1, *n* (%)	8 (8.0%)	13 (9.2%)	5 (5.3%)	
2, *n* (%)	25 (25%)	36 (26%)	31 (33%)	
3, *n* (%)	43 (43%)	64 (45%)	44 (46%)	
4, *n* (%)	19 (19%)	24 (17%)	14 (15%)	
5, *n* (%)	5 (5.0%)	4 (2.8%)	1 (1.1%)	

a Median (IQR); *n* (%).

b Kruskal-Wallis rank sum test; Pearson’s chi-squared test; Fisher’s exact test.

Abbreviations: ACS, acute coronary syndrome; CABG, coronary artery bypass graft; COPD, chronic obstructive pulmonary disease; EF, ejection fraction; GFR, glomerular filtration rate; HTA, artery hypertension; M, male; MI, myocardial infarction; *N*, number of patients; PAD, peripheral artery disease.

Postoperative outcomes (**Table 5**) showed no overall difference in complication rates.

**Table 5.** Postoperative Baseline Characteristics

Characteristic	Group 1	Group 2	Group 3	*P*-value^b^
	(*N* = 100)^a^	(*N* = 141)^a^	(*N* = 95)^a^	
Creatinine, mg/dL	0.81 (0.69, 0.95)	0.95 (0.84, 1.09)	1.44 (1.13, 1.94)	<0.001
Complication, *n* (%)	42 (42%)	64 (45%)	49 (52%)	0.4
Neurological events, *n* (%)	1 (2.0%)	5 (3.5%)	5 (5.3%)	0.5
Infective complications, *n* (%)	3 (3.0%)	5 (3.5%)	7 (7.4%)	0.3
Respiratory events, *n* (%)	18 (18%)	17 (12%)	20 (21%)	0.2
MI, *n* (%)	1 (1.0%)	1 (0.7%)	0 (0%)	>0.9
AF, *n* (%)	21 (21%)	37 (26%)	26 (27%)	0.5
AKI, *n* (%)	4 (4.0%)	2 (1.4%)	11 (12%)	0.002
IABP, *n* (%)	2 (2.0%)	4 (2.8%)	1 (1.1%)	0.9
Reoperation for bleeding, *n* (%)	1 (1.0%)	4 (2.8%)	3 (3.2%)	0.6
Mortality, *n* (%)	0 (0%)	1 (0.7%)	1 (1.1%)	0.7
Hospital stay, days	8 (5, 10)	7 (6, 9)	9 (7, 12)	<0.001

a Median (IQR); *n* (%).

b Kruskal-Wallis rank sum test; Pearson’s chi-squared test; Fisher’s exact test.

Abbreviations: AF, atrial fibrillation; AKI, acute kidney injury; IABP, intra-aortic ballon pump; MI, myocardial infarction; *N*, number of patients.

Group 3 had higher creatinine (*P* < .001) and more acute kidney injury (AKI) (12% versus 1.4% and 4.0% in Groups 2 and 1; *P* = .002), while atrial fibrillation and respiratory insufficiency were more frequent in Group 1. Hospital stay was longer in Group 3 (9 days [7-12] versus 7 days [6-9] and 8 days [5-10] in Groups 2 and 1; *P* < .001).

## DISCUSSION

In our study, we found that at the time of surgery, ITAs and SVGs used for the CABG did not exhibit significant differences in intimal thickening, fibroelastosis, or vasa vasorum density among patient groups stratified by kidney function. Venous grafts showed no differences in arterialization-related alterations, irrespective of renal impairment severity.

Subgroup analysis of CKD patients revealed no significant structural differences in ITAs and SVGs between those with GFR ≤59 mL/min and patients on dialysis, or between GFR of 58-31 mL/min and GFR ≤30 mL/min.

Clinically, patients with a GFR < 60 mL/min (Group 3) were older, had lower body mass, and had higher baseline creatinine and EuroSCORE II. Postoperatively, they had higher creatinine, greater AKI incidence, and longer hospital stays.

CKD is a well-established risk factor for vascular compromise, predominantly in arteries, and is associated with an increased atherosclerotic burden driven by mineral and bone disorders, inflammation, lipid abnormalities, and endothelial dysfunction.^1^^,^^2^^,^^4^^,^^5^^,^^7^^,^^22^^,^^28^ CKD is present in up to 19.4% of CABG patients, while coronary artery disease (CAD) affects 30% to 60% of dialysis population.^1^^,^^2^ Patients with CKD undergoing CABG are at elevated risk for postoperative complications, including AKI, prolonged hospitalization, and increased in-hospital mortality.^29^ Despite this, CABG remains the most effective revascularization strategy.^30^

CKD also affected the venous system, with documented intimal hyperplasia in dialysis access veins,^31–33^ and is implicated in SVG failure post-CABG.^8^^,^^13^

Despite the vascular burden in CKD, data on the structural state of grafts at surgery are limited.^3^^,^^9^^,^^15^^,^^18–22^

Our findings demonstrate that ITAs show minimal histological changes—such as intimal thickening or fibroelastosis—regardless of renal function. Most ITAs exhibited no intimal thickening, and fibroelastosis was negligible across all groups.

These findings reinforce the reliability of ITAs for CABG, even in end-stage CKD or dialysis patients, given their resistance to atherosclerotic remodelling.^8^^,^^13^^,^^17^^,^^21^^,^^22^

This durability is attributed to their unique anatomical and biological features, which appear preserved in CKD patients.^8–10^^,^^13^^,^^17^^,^^18^^,^^21^^,^^34–36^

As widely reported in the literature, SVGs exhibited greater intimal thickening than ITAs, reflecting their higher susceptibility to atherosclerosis.^9^^,^^10^^,^^13^^,^^15^^,^^16^^,^^31–33^^,^^37^^,^^38^

Ischaemia during harvesting and preparation induces oxidative stress and endothelial injury, promoting platelet adhesion.^9^^,^^10^^,^^13^^,^^15^^,^^16^^,^^31–33^^,^^37–39^ Activated platelets release mitogenic factors that drive smooth muscle migration and intimal hyperplasia, ultimately predisposing the graft to atherosclerosis and thrombosis.^9^^,^^10^^,^^13^^,^^15^^,^^16^^,^^31–33^^,^^37–39^

Most SVG cases were limited to Grade 1 thickening, with no significant differences across CKD groups—unlike the findings of Endo et al.^8^

Arterialization was uncommon, whereas fibroelastosis, likely reflecting chronic inflammation, was present in over half of SVGs irrespective of renal status.^15^ These results align with Fukushima et al., who showed that fibrointimal hyperplasia may develop within 1 month due to shear stress from arterial pressure, contributing to SVG arterialization.^9^^,^^13^^,^^15^

Interestingly, we observed no significant histological differences between dialysis patients and those with milder CKD. In contrast, other chronic conditions—such as Type 2 diabetes mellitus—have been shown to negatively affect structure and function, particularly in SVGs.^40^ Our study did not evaluate functional performance, and the interplay between CKD, age, and other factors remains unclear.^32^ Further research is warranted to confirm and expand upon our findings.

### Limitations

Limitations include the modest sample size, observational design, and absence of follow-up, providing only a “time-zero” histological snapshot. We did not differentiate between skeletonized and pedicled ITAs, nor capture CKD duration at surgery. Future prospective studies with functional and long-term follow-up (eg, angiography) are warranted.

## CONCLUSION

Unexpectedly, at the time of surgery, varying degrees of renal dysfunction—including dialysis—did not significantly affect the structural integrity of CABG conduits (arterial and venous), suggesting that these grafts may be suitable for use in this category of patients. Larger cohort and long-term studies are needed to assess the impact of CKD on graft performance and surgical outcomes.

## Supplementary Material

ivaf222_Supplementary_Data

## Data Availability

Data underlying this article will be shared upon reasonable request to the corresponding author.

## References

[1] Siddiqi S , Ravichandren K, Soltesz EG, et al Coronary artery bypass graft patency and survival in patients on dialysis. J Surg Res. 2020;254:1-6. 32388058 10.1016/j.jss.2020.03.069

[2] Giustino G , Mehran R, Serruys PW, et al Left main revascularization with PCI or CABG in patients with chronic kidney disease: EXCEL trial. J Am Coll Cardiol. 2018;72:754-765. 30092952 10.1016/j.jacc.2018.05.057

[3] Kay H , Korns M, Flemma R, et al Atherosclerosis of the internal mammary artery. Ann Thorac Surg. 1976;21:504-507. 1275603 10.1016/s0003-4975(10)63917-3

[4] Valdivielso JM , Rodríguez-Puyol D, Pascual J, et al Atherosclerosis in chronic kidney disease: more, less, or just different? Arterioscler Thromb Vasc Biol. 2019;39:1938-1966. 31412740 10.1161/ATVBAHA.119.312705

[5] Mathew RO , Bangalore S, Lavelle MP, et al Diagnosis and management of atherosclerotic cardiovascular disease in chronic kidney disease: a review. Kidney Int. 2017;91:797-807. 28040264 10.1016/j.kint.2016.09.049

[6] Sarnak MJ , Amann K, Bangalore S, et al; Conference Participants. Chronic kidney disease and coronary artery disease: JACC state-of-the-art review. J Am Coll Cardiol. 2019;74:1823-1838. 31582143 10.1016/j.jacc.2019.08.1017

[7] Tian L , Jaeger BC, Scialla JJ, et al; CRIC Study Investigators. Progression of coronary artery calcification and risk of clinical events in CKD: the chronic renal insufficiency cohort study. Am J Kidney Dis. 2025;85:67-77.e1. 39154888 10.1053/j.ajkd.2024.06.018 PMC12278985

[8] Endo D , Yamamoto T, Kajimoto K, et al Coronary artery bypass grafting in patients with chronic kidney disease: chronic kidney disease has an independent adverse effect on the long-term outcome of coronary artery bypass grafting. Biomed Res Int. 2022;2022:4994970. 35528157 10.1155/2022/4994970 PMC9071893

[9] Lytle BW , Loop FD, Cosgrove DM, et al Long-term (5 to 12 years) serial studies of internal mammary artery and saphenous vein coronary bypass grafts. J Thorac Cardiovasc Surg. 1985;89:248-258. 2857209

[10] Kraler S , Libby P, Evans PC, et al Resilience of the internal mammary artery to atherogenesis: shifting from risk to resistance to address unmet needs. Arterioscler Thromb Vasc Biol. 2021;41:2237-2251. 34107731 10.1161/ATVBAHA.121.316256 PMC8299999

[11] Marzouk M , Kalavrouziotis D, Grazioli V, et al Long-term outcome of the in situ versus free internal thoracic artery as the second arterial graft. J Thorac Cardiovasc Surg. 2021;162:1744-1752.e7. 32305200 10.1016/j.jtcvs.2020.03.003

[12] Garrett HE , Dennis EW, DeBakey ME. Aortocoronary bypass with saphenous vein graft. Seven-year follow-up. JAMA. 1973;223:792-794. 4567689

[13] Fukushima S , Kobayashi J, Niwaya K, et al Accelerated graft disease in a composite saphenous vein with internal thoracic artery in a chronic renal dialysis patient. Jpn J Thorac Cardiovasc Surg. 2004;52:372-374. 15384711 10.1007/s11748-004-0013-3

[14] FitzGibbon GM , Leach AJ, Keon WJ, et al Coronary bypass graft fate. J Thorac Cardiovasc Surg. 1986;91:773-778. 3486327

[15] Shelton ME , Forman MB, Virmani R, et al A comparison of morphologic and angiographic findings in long-term internal mammary artery and saphenous vein bypass grafts. J Am Coll Cardiol. 1988;11:297-307. 2892871 10.1016/0735-1097(88)90094-0

[16] Xenogiannis I , Zenati M, Bhatt DL, et al Saphenous vein graft failure: from pathophysiology to prevention and treatment strategies. Circulation. 2021;144:728-745. 34460327 10.1161/CIRCULATIONAHA.120.052163

[17] Kai M , Okabayashi H, Hanyu M, et al Long-term results of bilateral internal thoracic artery grafting in dialysis patients. Ann Thorac Surg. 2007;83:1666-1671. 17462376 10.1016/j.athoracsur.2006.12.010

[18] Sisto T , Isola J. Incidence of atherosclerosis in the internal mammary artery. Ann Thorac Surg. 1989;47:884-886. 2787973 10.1016/0003-4975(89)90027-1

[19] Sims FH. A comparison of coronary and internal mammary arteries and implications of the results in the etiology of arteriosclerosis. Am Heart J. 1983;105:560-566. 6837411 10.1016/0002-8703(83)90478-7

[20] van Son JA , Smedts F, Vincent JG, et al Comparative anatomic studies of various arterial conduits for myocardial revascularization. J Thorac Cardiovasc Surg. 1990;99:703-707. 2319794

[21] Ura M , Sakata R, Nakayama Y, et al The impact of chronic renal failure on atherosclerosis of the internal thoracic arteries. Ann Thorac Surg. 2001;71:148-151. 11216736 10.1016/s0003-4975(00)01700-8

[22] Kinoshita T , Asai T, Suzuki T, et al Histomorphology of right versus left internal thoracic artery and risk factors for intimal hyperplasia. Eur J Cardiothorac Surg. 2014;45:726-731. 23996658 10.1093/ejcts/ezt430

[23] Canham PB , Finlay HM, Boughner DR. Contrasting structure of the saphenous vein and internal mammary artery used as coronary bypass vessels. Cardiovasc Res. 1997;34:557-567. 9231039 10.1016/s0008-6363(97)00056-4

[24] Garnizone M , Vartina E, Pilmane M. Morphologic comparison of blood vessels used for coronary artery bypass graft surgery. Folia Morphol (Warsz). 2022;81:584-593. 34608982 10.5603/FM.a2021.0084

[25] Kdigo 2. Clinical practice guideline update for the diagnosis, evaluation, prevention, and treatment of chronic kidney disease-mineral and bone disorder (CKD-MBD). Kidney Int Suppl. 2011;7:1-59. 10.1016/j.kisu.2017.04.001 PMC6340919 30675420

[26] Milojevic M , Head SJ, Mack MJ, et al The impact of chronic kidney disease on outcomes following percutaneous coronary intervention versus coronary artery bypass grafting in patients with complex coronary artery disease: five-year follow-up of the SYNTAX trial. EuroIntervention. 2018;14:102-111. 29155387 10.4244/EIJ-D-17-00620

[27] Kwei S , Stavrakis G, Takahas M, et al Early adaptive responses of the vascular wall during venous arterialization in mice. Am J Pathol. 2004;164:81-89. 14695322 10.1016/S0002-9440(10)63099-4 PMC1602233

[28] Cai Q , Mukku VK, Ahmad M. Coronary artery disease in patients with chronic kidney disease: a clinical update. Curr Cardiol Rev. 2013;9:331-339. 24527682 10.2174/1573403X10666140214122234 PMC3941098

[29] Laimoud M , Alanazi MN, Maghirang MJ, et al Impact of chronic kidney disease on clinical outcomes during hospitalization and five-year follow-up after coronary artery bypass grafting. Crit Care Res Pract. 2023;2023:9364913. 37795473 10.1155/2023/9364913 PMC10547561

[30] Grazioli V , Di Mauro M, Perocchio G, et al Myocardial revascularization in patients with chronic kidney disease: a systematic review and meta-analysis of surgical versus percutaneous coronary revascularization. Interdiscip Cardiovasc Thorac Surg. 2025;40:ivaf021. 10.1093/icvts/ivaf021 PMC11897794 39969961

[31] Lee T , Chauhan V, Krishnamoorthy M, et al Severe venous neointimal hyperplasia prior to dialysis access surgery. Nephrol Dial Transplant. 2011;26:2264-2270. 21220751 10.1093/ndt/gfq733 PMC3145379

[32] Vazquez-Padron RI , Duque JC, Tabbara M, et al Intimal hyperplasia and arteriovenous fistula failure: looking beyond size differences. Kidney360. 2021;2:1360-1372. 34765989 10.34067/KID.0002022021 PMC8579754

[33] Monroy MA , Fang J, Li S, et al Chronic kidney disease alters vascular smooth muscle cell phenotype. Front Biosci (Landmark Ed). 2015;20:784-795. 25553479 10.2741/4337 PMC4331020

[34] Sons HJ , Godehardt E, Kunert J, et al Internal thoracic artery: prevalence of atherosclerotic changes. J Thorac Cardiovasc Surg. 1993;106:1192-1195. 8246559

[35] Grondin CM , Campeau L, Lespérance J et al Comparison of late changes in internal mammary artery and saphenous vein grafts in two consecutive series of patients 10 years after operation. Circulation. 1984;70:I208-I212. 6611220

[36] Chaikhouni A , Crawford FA, Kochel PJ, et al Human internal mammary artery produces more prostacyclin than saphenous vein. J Thorac Cardiovasc Surg. 1986;92:88-91. 3755199

[37] Ratliff NB , Myles JL. Accelerated atherosclerosis in saphenous vein bypass grafts—possible immune complex disease. Fed Proc 1984;43:844.

[38] Gharibeh L , Ferrari G, Ouimet M, et al Conduits’ biology regulates the outcomes of coronary artery bypass grafting. JACC Basic Transl Sci. 2021;6:388-396. 33997524 10.1016/j.jacbts.2020.11.015 PMC8093468

[39] Raza S , Chang C, Deo SV, et al Current role of saphenous vein graft in coronary artery bypass grafting. Indian J Thorac Cardiovasc Surg. 2018;34:245-250. 33060945 10.1007/s12055-018-0759-3 PMC7525697

[40] Lorusso R , Pentiricci S, Raddino R, et al Influence of type 2 diabetes on functional and structural properties of coronary artery bypass conduits. Diabetes. 2003;52:2814-2820. 14578301 10.2337/diabetes.52.11.2814

