# Supplementary material for: Histological Analysis of Arterial and Venous Grafts Used in Coronary Bypass for Patients With Renal Insufficiency: A Prospective Multicentre Observational Study
Source: Interdiscip Cardiovasc Thorac Surg. 2025 Sep 24;40(10):ivaf222. doi: 10.1093/icvts/ivaf222 (PMC12503227; doi:10.1093/icvts/ivaf222)
Supplement: ivaf222_Supplementary_Data [file ivaf222_supplementary_data.zip › Supplemental Material.docx]

**Supplemental Material**

Supplemental Figures Legends

Supplemental Figure 1. Histopathological changes explored in arterial grafts

A-D. Intimal thickening grading as 0 (A), 1 (B), 2 (C), and 3 (D).

E. *Vasa vasorum* in the *tunica adventitia*.

F. Fibroelastosis (pink/grayish material) within the arterial wall.

Symbols: Arrows indicate normal *tunica intima* (A); asterisk, *tunica media* (A-E); arrowheads, *tunica adventitia* (A-E); the double-headed arrows, intimal thickening (B-D); single arrow, intima (F).

Staining: Hematoxylin-eosin; magnification: x20 (scale bar = 200 µm).

Supplemental Figure 2. Histopathological changes explored in venous grafts.

A–D: Intimal thickening graded as 0 (A), 1 (B), 2 (C), and 3 (D).

E: Vasa vasorum in the *tunica adventitia*.

F: Arterialization with internal elastic lamina and wall thickening.

Symbols: Arrows indicate normal *~~null~~ tunica intima* (A); asterisks, *tunica media* (A–D, F); arrowheads, *tunica adventitia* (A–C, E–F); double-headed arrows, intimal thickening (B–D); single arrow, internal elastic lamina (F).

Stain: Hematoxylin-eosin; magnification: ×20 (scale bar = 200 µm).
