# Supplementary figures and images for: Histological Analysis of Arterial and Venous Grafts Used in Coronary Bypass for Patients With Renal Insufficiency: A Prospective Multicentre Observational Study
Source: Interdiscip Cardiovasc Thorac Surg. 2025 Sep 24;40(10):ivaf222. doi: 10.1093/icvts/ivaf222 (PMC12503227; doi:10.1093/icvts/ivaf222)

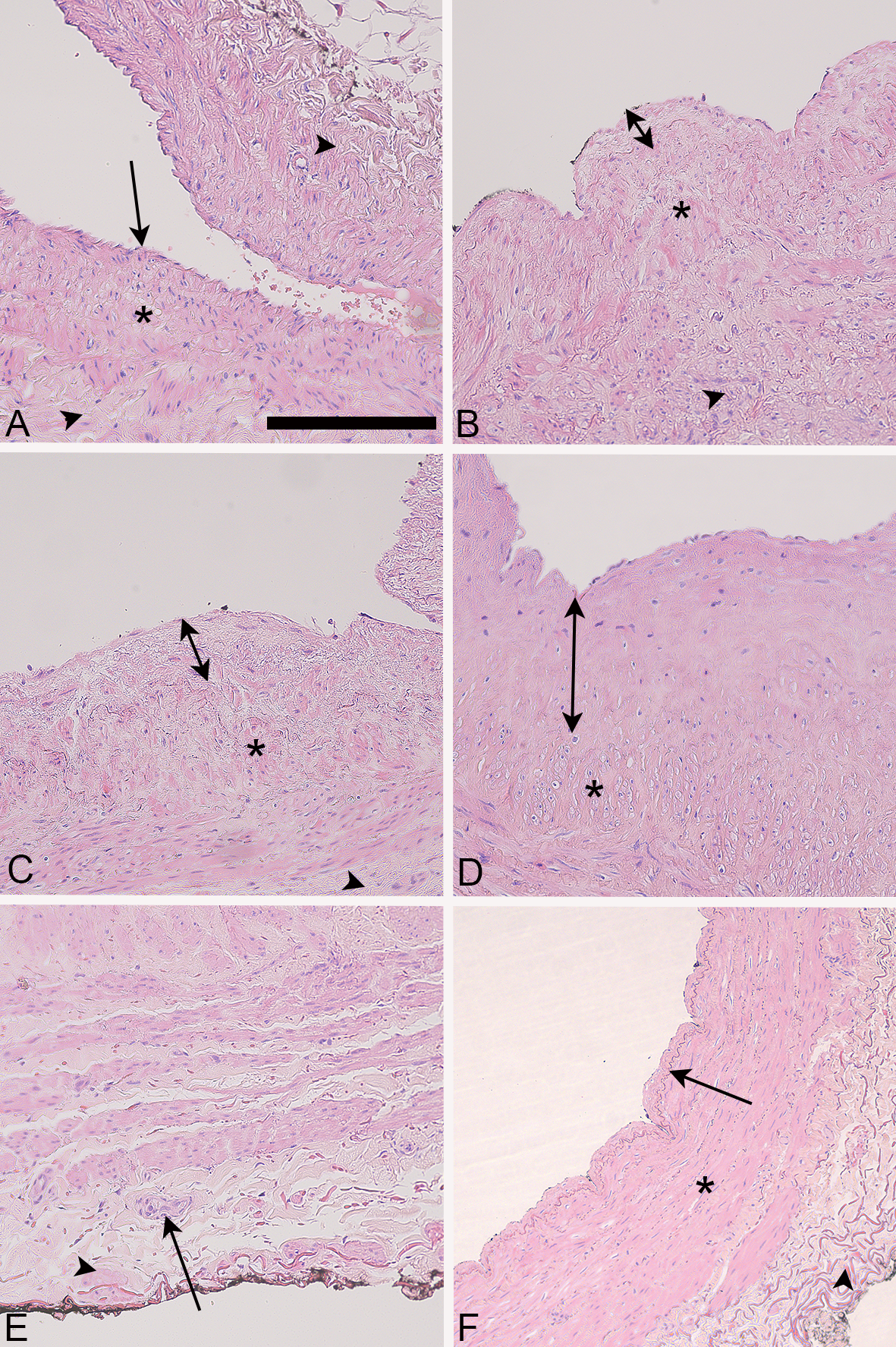

Supplement: ivaf222_Supplementary_Data [file ivaf222_supplementary_data.zip › Supplemental Figure 2.tif]

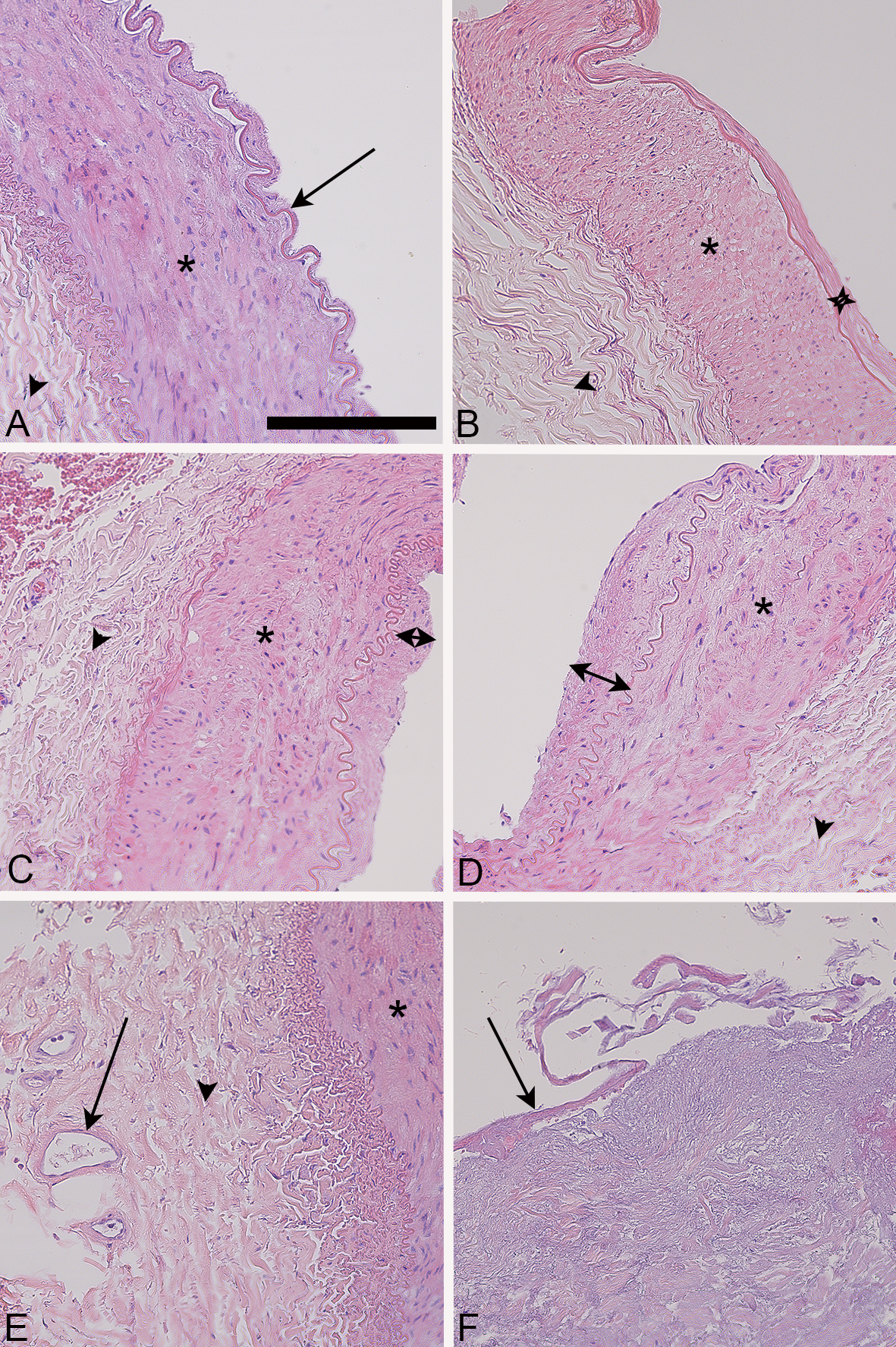

Supplement: ivaf222_Supplementary_Data [file ivaf222_supplementary_data.zip › Supplemental Figure 1.tif]
